# Source Apportionment and Geographic Distribution of Heavy Metals and as in Soils and Vegetables Using Kriging Interpolation and Positive Matrix Factorization Analysis

**DOI:** 10.3390/ijerph19010485

**Published:** 2022-01-02

**Authors:** Huiyue Su, Yueming Hu, Lu Wang, Huan Yu, Bo Li, Jiangchuan Liu

**Affiliations:** 1 College of Natural Resources and Environment, South China Agricultural University, Guangzhou 510642, China; huiyuesu@stu.scau.edu.cn (H.S.); ymhu@scau.edu.cn (Y.H.); 2 Guangdong Province Key Laboratory for Land Use and Consolidation, Guangzhou 510642, China; 3 Guangdong Province Engineering Research Center for Land Information Technology, Guangzhou 510642, China; 4 College of Earth Sciences, Chengdu University of Technology, Chengdu 610059, China; yuhuan0622@126.com; 5 South China Academy of Natural Resources Science and Technology, Guangzhou 510642, China; bli@ust.hk (B.L.); jcliu@cs.sfu.ca (J.L.)

**Keywords:** heavy metal, soil, vegetable, source apportionment, positive matrix factorization model

## Abstract

Food security and cultivated land utilization can be seriously affected by heavy metal (HM) pollution of the soil. Therefore, identifying the pollution sources of farmland is the way to control soil pollution and enhance soil quality effectively. In this research, 95 surface soil samples, 34 vegetable samples, 27 irrigation water samples, and 20 fertilizer samples were collected from the Wuqing District of Tianjin City, China and was used to determine their HMs accumulation and potential ecological risks. Then, kriging interpolation and positive matrix factorization (PMF) were utilized to identify the sources of soil HMs. The results indicated that soil HMs in the study area were contaminated at a medium level, but that the pollution of Cd was more severe, and the Cd content in vegetables was slightly higher than the permissible threshold (0.02 mg·kg^−1^). Furthermore, a non-homogeneous distribution was observed, with higher concentrations of HM contaminants concentrated in the southwest of the study area, where many metal manufacturing industries are located. Our results suggest that the Cd originated from industrial activity; As and Pb from agricultural practices; Ni, Cu, Cr, and As mainly from natural sources; Zn and Cu from organic fertilizer; Pb and Cd mainly from traffic discharge; and Cr, Ni, and Pb from sewage irrigation. Obviously, the accumulation of soil HMs in the study area could be mainly attributed to industrial activities, implying the need for implementation of government strategies to reduce industrial point-source pollution.

## 1. Introduction

Intensive industrial and agricultural activities during rapid industrialization and urbanization lead to the accumulation of heavy metals (HMs) in soil through atmospheric deposition, sewage irrigation, and other routings [1,2]. A high accumulation of HMs in the soil would not only reduces crop yields and quality, but also pose a severe threat to the ecosystem and human security [3,4]. However, HM pollutants in the soil of urban-rural fringe areas may come from a variety of sources, including natural and anthropogenic sources (such as atmospheric deposition, waste incineration, sewage irrigation, fertilizer application and pesticide application) [5,6,7]. Thus, determination of HM sources in soil is very important for food security and the sustainable utilization of cultivated land [8,9].

Stochastic models and receptor models are the two main tools for HM identification in soil [10,11,12]. Stochastic models are regarded as essential methods for source identification, which are based on the statistical analysis of sampled data along with corresponding environmental parameters [13]. Nevertheless, stochastic models are highly dependent on prior knowledge about source profiles. A lack of such knowledge will lead to inaccurate results [14].

There are also many problems with receptor models [15,16]. The models are mainly used for evaluating the contributions of each pollution source. They are dependent on observations of environmental samples (the “receptors”) regardless of any knowledge about all pollution sources [17,18,19]. The most widely used receptor models include principal component analysis/absolute principal component scores (PCA/APCS), positive matrix factorization (PMF), and UNMIX [20,21]. For PCA/APCS, the source contributions are derived based on regressions between HM concentrations and APCS [22]. Often, the results may have unreasonable negative contributions [23]. UNMIX is a non-negative constraint factor analysis method developed using the self-modeling curve resolution (SCMR) technique [24]. It has been often used to determine the source distribution of HMs in atmospheric particles and sediments [25,26]. The PMF is a matrix decomposition method using non-negative constraint conditions, which can manipulate missing and inexact data. As such, it is a simple and feasible source analysis method [27,28].

Further, the receptor model is subjective in the identification of pollution sources [23], and it regards the pollutant propagation process as a linear process [29]. Therefore, it ignores important information contained in spatial correlation between environmental samples.

On the other hand, geostatistics with stochastic simulation could provide detailed spatial information about spatial characteristics of HM [30]. Thus, it can be used to explain results of a receptor model by mining information from the spatial characteristics [17,21]. Thus, a combination of geostatisics with a receptor model can objectively identify pollution sources and quantify their contribution rate.

This study proposes such a method of combining ordinary kriging interpolation with PMF analysis, for source apportionment of HM in soil. Specifically, the three main objectives are as follows: (1) determine the spatial distribution characteristics of HMs in an urban fringe area; (2) assess the accumulation of HMs in vegetables and the factors affecting HM accumulation; (3) analyze the possible pollution sources of farmland soil and quantify the contribution rate of pollution sources by ordinary kriging interpolation combined with PMF.

## 2. Materials and Methods

### 2.1. Study Area

The study area is in Wuqing, northwestern Tianjin (116°46′–117°19′ E, 39°07′–39°42′ N), which is located in the lower part of the North China alluvial plain, with a gentle terrain and a total area of 1574 km^2^. The region features a temperate continental monsoon climate, with an annual average temperature of 11.6 °C, and an annual rainfall of 500–610 mm. Northwest winds are prevailing throughout the year, and annual average wind speeds are about 2–4 m·s^−1^. There are abundant high-quality coal reserves in the northwestern of Wuqing. The area also has many industrial parks, including industry, material manufacturing, automobile parts industry, and steel industry. It is also one of the main vegetable production areas in Tianjin city. Soil types in the area include fluvo-aquic soil, loam fluvo-aquic soil, sandy fluvo-aquic soil, clay fluvo-aquic soil, salinized fluvo-aquic soil, humid fluvo-aquic soil, and limestone leaching fluvo-aquic soil in terms of the classification and codes for Chinese soil (GB/T17296-2009). As a typical urban fringe area, the agriculture, industry, transportation, and other human activities in Wuqing District have changed significantly during the past decades. Thus, the characteristics of HM pollution sources in farmland are complex, seriously threatening the sustainable use of regional agricultural resources and the safety of agricultural products.

### 2.2. Data Acquisition

**Figure 1:**
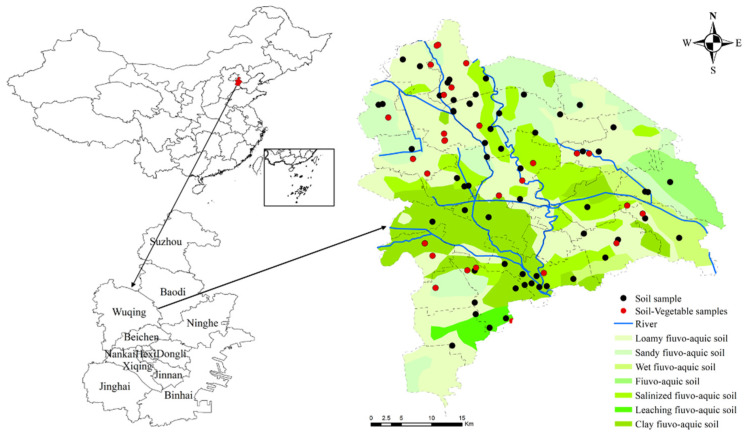
Location of the study area and the sampling point distribution.

The cultivated land in this study area is 912.92 km^2^, accounting for 58% of the total area. The vegetable land area is 233.34 km^2^, accounting for 25.56% of the cultivated land area. Due to the low degree of adjacent vegetable fields, the grid sampling method cannot ensure vegetable planting in each grid. Therefore, this study adopts the route survey method to collect the samples with the same type of vegetable planting, considering different fertilizers. A total of 95 surface top soil samples (bulk soil), 34 vegetable samples, 20 organic fertilizer samples, and 27 irrigating water samples were collected. The spatial distribution of the samples is shown in Figure 1.

#### 2.2.1. Soil and Vegetable Sample Collection

Soil samples (0–20 cm) were collected according to the standard sampling procedure (HJ/T166-2004). Each sample was composed of five subsamples, and the latitude and longitude coordinates of the sampling points were recorded during sampling. Thirty-four vegetable samples were collected from the same locations as soil samples. The samples were immediately preserved in polythene bags and dried at 60 °C for two hours Roots and other plant parts were separated and stored in moisture-proof plastic bags. To remove impurities such as weeds, roots, and gravels, air-dried soil samples were ground to pass through a 2-mm screen. Further, the samples were ground to pass through a 100-mesh nylon sieve and then stored in polyvinyl chloride (PVC) bags for analysis.

#### 2.2.2. Collection of Irrigation Water and Organic Fertilizer

Twenty-seven irrigation water samples were collected from local surface runoff (HJ/T-91-2002). Before analysis, samples were stored in coolers to minimize biodegradation and volatilization.

Organic fertilizer (e.g., cow manure, chicken manure, and pig manure) was collected and stored in PVC bags before analysis.

#### 2.2.3. Analytical Method

The soil and organic fertilizer samples were digested with a mixture of HCl-HNO_3_-HF-HClO_4_, while vegetable and irrigation water samples were digested with a mixture of HNO_3_-H_2_O_2_. The total HM (Pb, Cu, Cr, Ni, and Zn) content in the soil, organic fertilizer, and irrigation water samples were determined using a flame atomic absorption spectrophotometry (Analytik Jena novAA 350), the total HM (Pb, Cu, Cr, Ni, and Zn) content in vegetable samples was determined using an inductively coupled plasma mass spectrometer (Agilent 7900 ICP-MS). The total Cd and total As in the soil, vegetable, organic fertilizer, and irrigation water samples were determined using a graphite furnace atomic absorption spectrometer (Analytik Jena ZEEnit 650P) and an atomic fluorescence spectrometer (Beijing Jitian, AFS-933), respectively. The DTPA—extractable metals were measured using an inductively coupled plasma mass spectrometer (Agilent 7900 ICP-MS). Soil pH was determined by a pH meter according to the ratio of water to soil 2.5:1 (*v*/*w*). Soil organic matter (SOM) was determined by the potassium dichromate oxidation-external heating method. For the determination of total metal content, the certified reference soil (GSS-16) was used as the quality control. The recoveries of HM elements in the reference sample ranged from 91–107%.

### 2.3. Evaluation of HMs Pollution in Soil

The geoaccumulation index ($I_{geo}$) and pollution load index (*PLI*) are common methods for quantitatively evaluating the HMs pollution of soil.

$I_{geo}$ is used to assessment the pollution status of HMs in soil by comparing the test concentration in soil with the natural geochemical background value, and *PLI* can directly reflect the contribution of each HM to the combined pollution of all HMs. $I_{geo}$ and *PLI* were determined as follows [31].

(1) $$I_{geo}=\log_{2}\left(\frac{C_{n}^{x}}{k\times C_{b}^{n}}\right)$$

(2) $$PLI={\left(\frac{C_{1}^{x}}{C_{b}^{1}}\times \frac{C_{2}^{x}}{C_{b}^{2}}\times \ldots \times \frac{C_{n}^{x}}{C_{b}^{n}}\right)}^{\frac{1}{n}}$$

where $C_{n}^{x}$ is the measured concentration of HM *n* (*n* = 7) in sample *x* (mg·kg^−1^), $C_{b}^{n}$ is the background concentrations of HMs in soils reported by Fan and Zhang [32] (i.e., 8.39, 0.09, 63.69, 19.88, 26.69, 20.6, and 66.87 mg·kg^−1^ for As, Cd, Cr, Cu, Ni, Pb, and Zn, respectively), and *k* is a parameter accounting for possible differences in the background values due to lithological variations. In this study, *k* was equal to 1.5. The $I_{geo}$ and *PLI* assessment standards of HMs as shown in Table 1.

### 2.4. Ecological Risk Assessment of HMs in Soil

The potential ecological risk index (*PERI*) is used to evaluate the ecological risk of HMs based on the content and toxicity of HMs in soil, reflecting the comprehensive effect of multiple pollutants and determines the potential degree of harm using a quantitative method [33]. The *PERI* was determined as follows:

(3) $$E_{r}^{i}=T_{r}^{i}\times \left(\frac{C_{x}^{i}}{C_{b}^{i}}\right)$$

(4) $$RI=\sum_{i}^{n}T_{r}^{i}\times \frac{C_{x}^{i}}{C_{b}^{i}}$$

where $E_{r}^{i}$ is the single *PERI* of an individual metal (*i*), *RI* is the *PERI* of sampling sites, $T_{r}^{i}$ is toxic response factor for HMs (*i*), which were As = 10, Cd = 30, Cu = Pb = 5, Zn = 1, Cr = 2, and Ni = 5 [33]. The *PERI* assessment standards of HMs as shown in Table 1.

### 2.5. Bioconcentration Factor

Bioconcentration factor (*BCF*) is an important method for studying the migration and transformation of HMs from soil to crops. The BCF can reflect the ability of crops to absorb and become enriched with metals from soil [34]. The *BCF* values were determined as follows:

(5) $$BCF=\frac{C_{plant}^{x}}{C_{soil}^{x}}$$

where $C_{plant}^{x}$ is the concentration of HMs in crops (mg·kg^−1^) and $C_{soil}^{x}$ is the concentration of HMs in soil (mg·kg^−1^).

### 2.6. Positive Matrix Factorization Model

The PMF model is dependent on the least-square algorithm for iterative calculation, and the original matrix X is decomposed continuously, to obtain the optimal matrices G and F. The optimization goal is to make Q values be as small as possible. The error of the receptor chemical composition is determined by the weight, and the main pollution sources and contribution rate is determined by the least-square method [35]. The matrix of specific sample data is decomposed into two matrices, factor contribution G (*i* × *k*) and factor distribution *F* (*k* × *j*), and a residual matrix *E* (*i* × *j*).

(6) $$X_{ij}=\sum_{k=1}^{p}G_{ik}F_{kj}+E_{ij}$$

In the formula, $X_{ij}$ is the concentration of the *j*th chemical component of the *i*th sample; *p* is the number of factors; $G_{ik}$ is the contribution of source *k*th to the *i*th sample (i.e., the sharing rate matrix of the source); $F_{kj}$ is the concentration of the *j*th chemical component in the source *k* (i.e., the source component matrix); and $E_{ij}$ is the residual matrix.

PMF defines an objective function Q:

(7) $$Q=\sum_{i=1}^{n}\sum_{j=1}^{m}{\left(\frac{e_{ij}}{u_{ij}}\right)}^{2}$$

In the formula, $u_{ij}$ represents the uncertainty of the *j*th chemical composition of the *i*th sample.

In the PMF model, concentration data and uncertainty data are needed. The calculation method of uncertainty data is as follows:

If the content of each element is less than or equal to the method detection limit (*MDL*), the uncertainty value is:

(8) $$U_{nc}=\frac{5}{6}\times MDL$$

Otherwise, the uncertainty value is:

(9) $$U_{nc}=\sqrt{{\left(\sigma \times c\right)}^{2}+MDL^{2}}$$

where σ is the relative standard deviation; *c* is the element concentration; and *MDL* is the method detection limit.

### 2.7. Statistical and Geostatistical Analysis

The content of HMs in soil was analyzed by descriptive statistical analysis (maximum, minimum, average, and standard deviation). Pearson tests were applied to determine the relationships between soil total/available metal content, pH, SOM, and metal accumulation in vegetables. In addition, Kolmogorov-Smirnov (K-S) tests were used to determine whether the concentration of HMs conformed to a normal distribution. Concentration data (Zn, Cd) that did not conform to a normal distribution were standardized by logarithmic transformation. Ordinary kriging was used to draw the spatial distribution map of HMs in soil.

## 3. Results

### 3.1. Accumulation of HMs in Soil

Descriptive statistics for soil pH, SOM, and total HMs in farmland of the study area are shown in Table 2. The average pH of the soil was 7.69 (6.63–8.89), and the pH of 4.21% of soil samples was lower than 7.0. The content of SOM ranged from 7.17 to 42.48 g·kg^−1^, with an average value of 19.30 g·kg^−1^, which is similar to the average content of SOM in the soil in China (19.8 g·kg^−1^) [36].

The average Zn, Cr, Pb, Cu, Ni, As, and Cd concentrations in soil (Table 2) indicate that the soil is polluted with Cd. Compared to the risk screening values for soils, the overall exceedance rates for Cd, Pb, As, Cu, and Zn were 5.26%, 2.11%, 1.05%, 2.11%, and 2.11%, respectively. The occurrence of exceedances in As and Pb contamination (1.59% and 3.16%) was in soil with a pH over 7.5, and exceedances for Cu (6.25%) and Zn (6.25%) occurred in medium acid soil (6.5 < pH < 7.5). The exceedance rates for Cd in neutral soil (6.5 ≤ pH ≤ 7.5) and alkaline soil (pH > 7.5) were 6.25% and 3.16%, respectively. The coefficient of variation (CV) indicates that the spatial distribution of metals Zn, Pb, Cu, and Cd was non-homogeneous [31]. Based on K-S and Shapiro-Wilk tests, the soil Cr, Pb, Cu, Ni, and As content was normally distributed.

**Figure 2:**
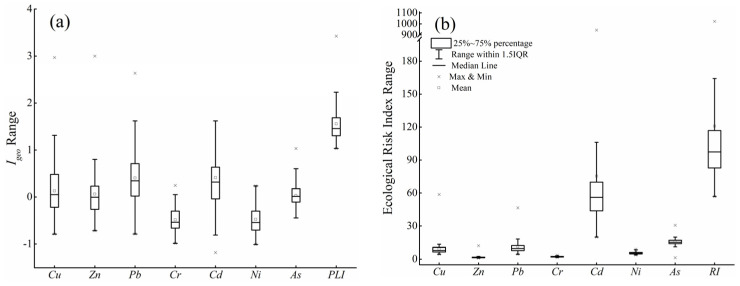
Geo-accumulation index ($I_{geo}$, **a**) and ecological risk index (**b**) of HMs.

$I_{geo}$ and *PLI* are shown in (Figure 2). The average $I_{geo}$ values were highest for Cd (0.41), followed by Pb (0.40), Cu (0.13), Zn (0.06), As (0.03), Ni (−0.48), and Cr (−0.49), indicating that the uptake of Cd, Cu, Pb, and Zn was higher in soils compared with other metals. More than 69.47%, 76.84%, 54.74%, and 48.42% of the samples were polluted with Pb, Cd, Cu, and As, with $I_{geo}$ ranging from the medium value (i.e., 1) to the high levels of polluted (i.e., 5). In addition, 85.26% and 14.74% of the samples were at the levels of medium (1 < *PLI* ≤ 2) to highly polluted (2 < *PLI* ≤ 5).

The *PERI* of HMs in soil ranged from 0.91 to 942.67 (Figure 2), indicating that HMs accumulated in the soil poses risks to local ecosystems. A total of 1.05%, 2.10%, and 9.47% of soil samples had extreme, strong, and moderate potential ecological risks, respectively. The individual index values were highest for Cd, followed by As, Pb, Cu, Ni, Cr, and Zn, which was similar to the pattern of $I_{geo}$ values. Cd, As, Pb, and Cu were the dominant HMs in soil posing potential risks, and Cd was the main factor causing ecological risks.

### 3.2. Bioavailability of HMs in Soil

**Figure 3:**
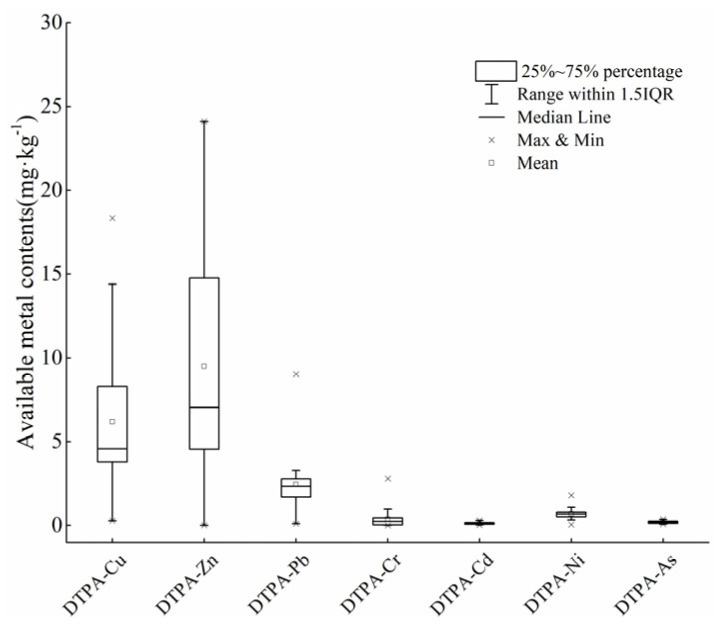
DTPA-extractable metals content (mg·kg^−1^) in soil.

In addition to the total HMs, speciation of HMs is also key for evaluating the degree of pollution, because the accumulation of HMs in vegetables is usually positively correlated with the availability of metals in soil [31]. The DTPA-extractable metal content in soil is shown in Figure 3. The average DTPA-Pb, Cd, Cu, As, Zn, Ni, and Cr content in soil was 2.46, 0.12, 6.20, 0.19, 9.50, 0.71, and 0.38 mg·kg^−1^, respectively.

**Figure 4:**
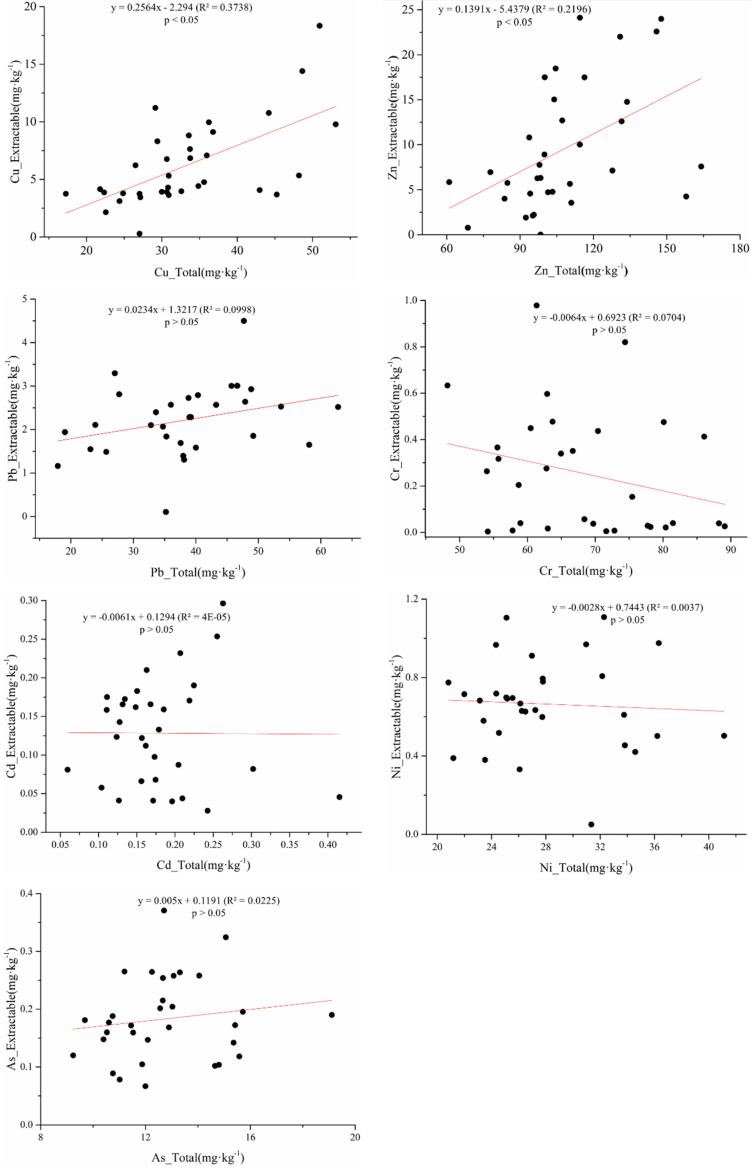
The available metal content (mg·kg^−1^) versus the total metal content (mg·kg^−1^) in soils.

The soil DTPA extraction rates of Pb, Cd, Cu, As, Zn, Ni and Cr were about 6.68%, 74.46%, 18.22%, 2.34%, 8.51%, 2.61% and 0.57%, respectively. In addition, the total metal contents of Cu and Zn were positively correlated with the DTPA-extractable metal contents (Figure 4). However, there was no correlation among the DTPA - As to total As content, which may be due to the properties of DTPA (such as high avidity of metal cations). These findings are similar to the results of a previous study showing the DTPA-extractable concentration in soils were significantly correlated with the total concentrations of Zn, Cu, and Pb, but not with As [37].

**Figure 5:**
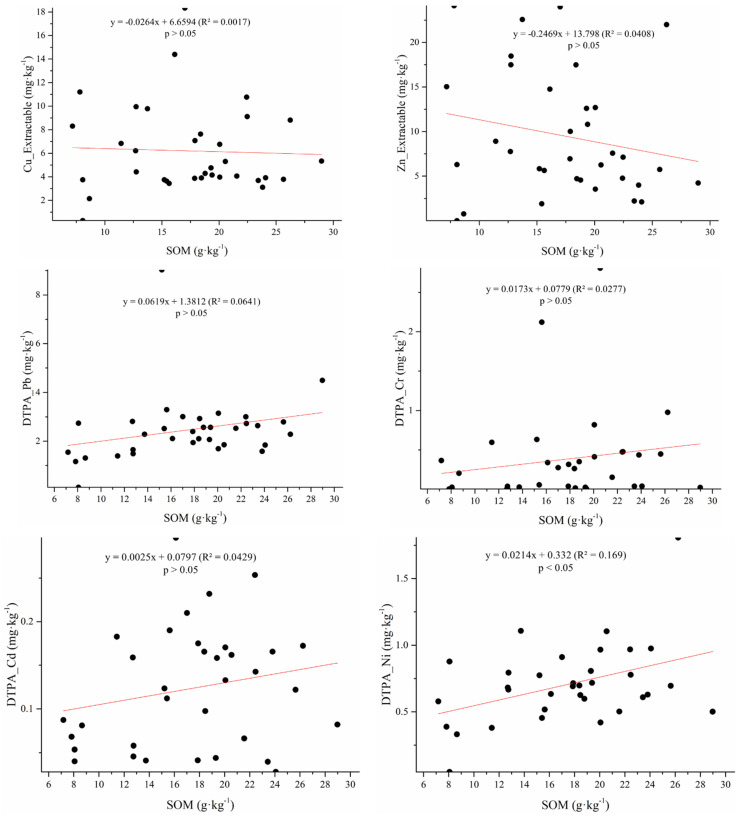

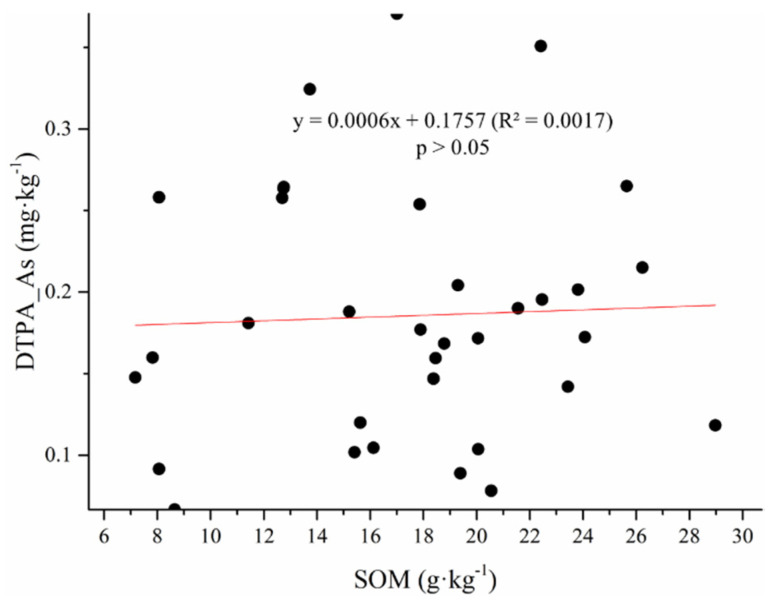
DTPA-extractable metal content (mg·kg^−1^) versus soil organic matter content (g·kg^−1^).

Available Cr, Pb, Ni, Cd, and As were directly correlated with the SOM content (Figure 5), demonstrating that the logarithm of DTPA-extractable metals (Pb, Cr, Mn, or Fe) was positively related to the SOM content, similar to a previous study [38]. However, a negative correlation between available HMs and the SOM content has also been reported [39]. This might be due to different compositions and properties of SOM in the studies.

### 3.3. Spatial Distribution of Soil Metals

To identify hot spots and potential sources of HMs in soil, spatial variation was assessed using ordinary kriging. The assessment had a mean error close to 0, and a root mean square standard error between 0.980 and 1.003, suggesting that the ordinary kriging prediction results were accurate.

**Figure 6:**
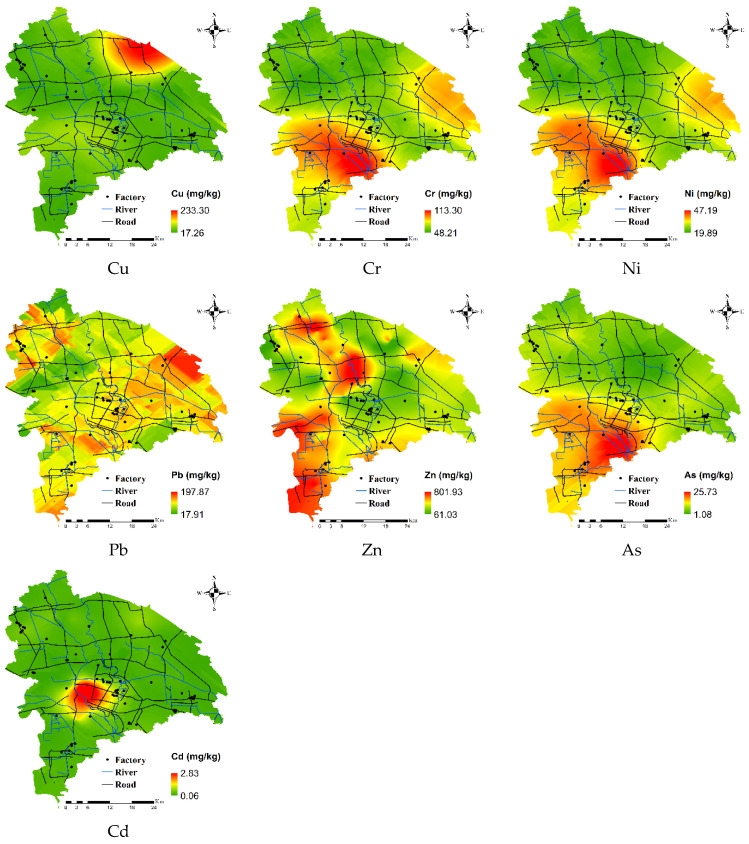
Spatial distribution of HMs in the study area.

The prediction results of HMs (Figure 6) shows that the spatial distribution of Cd was relatively high in the southwest, and the high values in this region indicate point source pollution. According to the results of a Google Earth query, there are 10 HM pollution enterprises in this region. The primary types of enterprises were electroplating, electronics, and chemicals, while industrial activities were very high (e.g., alloy processing). Excessive industrial activity is one of the main causes of Cd accumulation [40]. High values of Pb were due to main roads with high traffic; automobile exhaust contains a large amount of Pb, which is commonly considered as an indicator of vehicular transport [41,42].

Variation in Cr, Ni, and As was highly consistent; in the mid-lower reaches of the river in the region, areas with high values of Cr, Ni, and As corresponded to the clay fluvo-aquic soil area, indicating that soil type was the main factor affecting the distribution of these HMs [43]. Parent materials might be primary sources of Cr, As, and Ni. Cu pollution was relatively concentrated in the northeast. Agricultural facilities in the region have undergone rapid growth, and this region has become the main area for vegetable planting. Measurements of the HM content of organic fertilizer samples in the study area revealed a large amount of Cu (Table 3). Thus, year-round fertilization and irrigation has lead to the enrichment of Cu and non-point source pollution; Cu should thus be present in organic fertilizer [44,45]. Zn in the soil was mainly in the southwest and northwest parts of the study region. Zn and Pb have similar spatial differentiation characteristics, and areas with the highest Zn and Pb values were close to main roads and rivers with high traffic. Automobile parts (e.g., brake pads, fuel tanks, and pads) and mechanical wear of automobile parts release Zn into the environment [19,46]. Therefore, traffic emissions might be the main reason for Zn enrichment.

### 3.4. Accumulation of HMs in Vegetables

**Figure 7:**
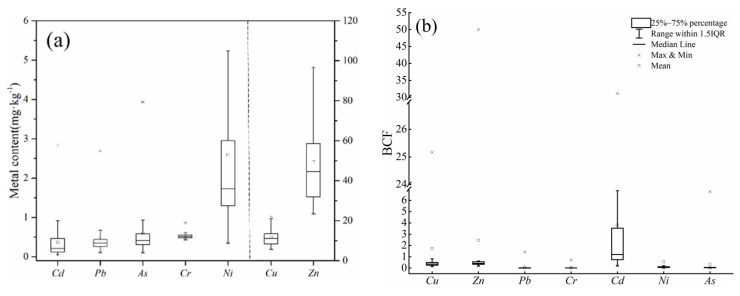
(**a**) Metal accumulation in vegetable samples and (**b**) bioconcentration factor (BCF) of different metals.

The concentrations of HMs in vegetable samples are shown in Figure 7. The average Cd, Pb, As, Cr, Cu, Ni, and Zn concentrations were 0.38, 0.46, 0.60, 0.53, 11.65, 2.58, and 48.97 mg·kg^−1^, respectively. The accumulation of Cd, Pb, As, and Cr in vegetables was slightly above the permissible threshold of 0.2, 0.3 0.5, and 0.5, respectively (GB2762-2017). The exceedance rates of Cd, Pb, As, and Cr in vegetable samples were 52.94%, 55.88%, 41.18%, and 58.82%, respectively. In general, Cd and Pb were the primary metals causing vegetable pollution. These results were similar to pervious research, but not exactly consistent. Chen et al. [47] proved that Cd pollution is most severe in vegetable-planting areas, followed by Pb and Cu. Cd enrichment in vegetable samples explains why the growth of leafy vegetables was affected by DTPA-Cd in soil. As concentrations of the HMs in soil increased, the concentrations of HMs in the edible parts of leafy vegetables also increased.

The biological concentration factor (*BCF*) of HMs in vegetables can be used to evaluate the ability of vegetables to absorb HMs. Smaller BCFs correspond to a higher resistance of vegetables to HM pollution in soil [48]. The concentrations of HMs in vegetables are shown in Figure 7. The pattern of BCF values differed from the pattern of bioavailable metal content. Specifically, BCF values were the highest for Cd, followed by Zn, Cu, Ni, As, Pb, and Cr, with average values of 2.47, 0.47, 0.37, 0.09, 0.08, 0.01, and lower than 0.01, respectively. Although Cd did not have a very high available metal content in soils, vegetables were highly enriched with Cd, consistent with the findings of previous studies [49].

**Figure 8:**
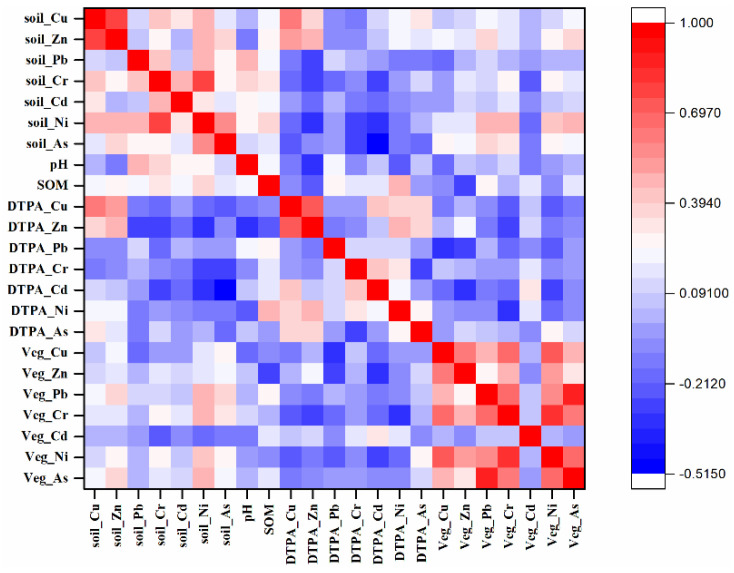
Pearson correlation matrix of HMs in soils (mg kg^−1^), soil DTPA-extractable metal content (mg kg^−1^), and metal accumulation in vegetable samples (mg kg^−1^).

Pearson correlations between HMs concentrations in vegetables and soils are shown in Figure 8. There was no significant relevance between the accumulation of HMs in vegetables and the content of HMs in soil, which was consistent with previous studies [50,51]. The weak correlation and lack of a linear relationship may be related to different forms of HMs in soil and their different bioavailabilities to vegetable crops, explaining why the Cd content was lower in soil but higher in vegetables [49]. Generally, assessments of soil metal pollution should consider the availability of HMs, rather than solely soil total metal content.

Soil properties also affected HM accumulation in vegetables. In this study, the SOM content was positively related to the accumulation of Pb, Cr, As, and Cd, but was negatively correlated with the accumulation of Zn, Cu, and Ni in vegetables (*p* < 0.05). The cumulative increase in Pb, Cr, and Cd concentrations in vegetables within soil samples having higher SOM can be explained by the increasing contents of available forms of HMs (Figure 5). Overall, as the SOM content in soil increased, the available content of Pb, Cr, and Cd in soil increased, and vegetable roots more easily absorbed Pb, Cr, and Cd in soil [52]. Thus, higher SOM resulted in the decrease of Cu and Zn in vegetables, which might stem from a decrease of available HMs in soil caused by pH and SOM. However, studies have proved that not all forms of Cu and Zn can be absorbed by vegetables, and the introduction of organic fertilizer residues of stable carbon compounds (such as humic substances) by adsorption or complexation further leads to low utilization of heavy metals [34]. Furthermore, atmospheric deposition of concentrated metallic particles on vegetable leaves may also be caused by the accumulation of HMs, because leaf absorption is a potential pathway for HM accumulation in vegetable [53].

### 3.5. Source Identification of Metals in Soils

**Figure 9:**
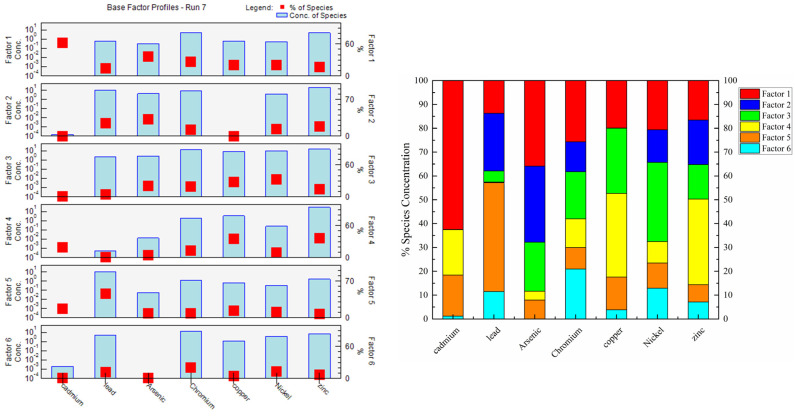
Source profiles from the PMF model and contribution of different factors to HM accumulation.

Six sources of HMs were identified by source apportionment using PMF shown in Figure 9, which was dependent on minimization and stable Q value to determine the most suitable number of factors. Residuals of most soil samples ranged from −3.0 to 3.0, and R^2^ > 0.94 indicating a strong correlation between HMs. The PMF results are shown in Figure 9.

The first source (S1) was characterized by Cd, with loading values over 27.8%. Anthropogenic Cd in soils may be derived from atmospheric deposition, industrial wastewater, and organic fertilizer [54]. Then, we analyzed the Cd content in organic fertilizer (Table 3) and irrigation water (Table 4) to determine whether Cd pollution was related to organic fertilizer and industrial wastewater. The Cd content in irrigation water did not exceed the maximum allowable metal content (GB5084-2005), and the Cd content in organic fertilizer was between 0.04 and 0.59 mg·kg^−1^. According to the organic fertilizer industry standard (NY525-2012), the Cd content in organic fertilizer samples did not exceed the standard (Table 3). Many studies have suggested that atmospheric deposition is the main source of soil Cd accumulation [55,56]. Given that the spatial variation of Cd was consistent with point-source pollution rather than non-point source pollution (Figure 6), Cd may have been derived from industrial sources. Therefore, S1 is attributed to industrial sources.

The second source (S2) was dominated by As and Pb (31.9% and 24.2%, respectively) and was interpreted as the utilization of agro-chemicals (i.e., pesticides/herbicides). Excessive application of agricultural substances such as pesticides, fertilizers, and plastic film can lead to HM pollution in farmland. Pesticides containing As, Hg, and Pb have been banned in most countries; however, there is still a large amount of As and Pb accumulation in soil because these metals are difficult to degrade and have been widely used to make pesticides such as insecticides and herbicides, which leads to their accumulation in soil [57,58]. Considering the application rate of Cd (40–50 kg·ha^−1^·a^−1^), as much as 2.74 μg·kg^−1^ soil a^−1^ Cd can be introduced into surface soils annually via fertilizer application. Pb has mainly been used to make heat stabilizers in agricultural plastic film mulching. Analysis of the Pb content in soil and plants with different residual films has shown that the Pb content is higher in soil and plants with high residual film than in soil and plants with low residual film [59]. Therefore, S2 corresponds to a mixture of pesticides, fertilizers, and plastic films.

The third source (S3) was mainly related to Ni, Cu, Cr, and As (33.2%, 27.4%, 19.7%, and 20.5%, respectively), which indicated the close relationships among these metals and their common sources. Many studies have demonstrated that Ni, Cr, and Cu are usually considered indicators of natural sources. For example, multivariate statistical analysis of the agricultural soil in the Isfahan Industrial Zone of Iran has shown that the main sources of Ni, Co, Cr, Fe, and Al are from genesis inputs [60]. Similar conclusions have also been obtained in Pastoriza [61], Huizhou [11], Isfahan [60], the Yellow River Basin [62], and Pernambuco [63]. Furthermore, the average concentrations of Ni, Cu, Cr, and As were all lower than their background values; according to $I_{geo}$ and *PLI*, small amounts of Cu and As can be harmful to the environment, which also stemmed from the long-term use of pesticides and livestock manure and the enrichment of Cu and As in soil. This conclusion is consistent with S1 and S4. Therefore, S3 represents a natural source.

The fourth source (S4) was related to the accumulation of Zn and Cu in soils (35.9% and 35.1%, respectively). Zn and Cu are transferred to animal feces as livestock daily feed additives because of the low utilization rate of metals by animals. Thus, Zn and Cu were usually used as markers of livestock manure [44,45]. In addition, organic fertilizer contained high concentrations of Zn and Cu, which also confirms that S4 corresponded to organic fertilizer (Table 3).

Pb (45.7%) and Cd (17.3%) were associated with the fifth source (S5). Automobile exhaust emissions are the main route by which Pb enters the soil, therefore traffic emissions are considered to be the main source of Pb pollution [64,65]. Although China has banned the use of leaded gasoline since 2000, the Pb content in roadside soils is still high as reported by a number of recent studies [4]. Cadmium powder produced by automobile fuel combustion and tire wear can also cause cadmium accumulation in soil [66]. The concentrations of Cd and Pb tended to decrease with increased distance from the road [4]. The spatial distribution characteristics indicate that high values of these two metals were concentrated near main roads, such as expressways, national highways, and provincial roads in the study area. In summary, S5 could be interpreted as traffic emissions.

The sixth source had a high factor loading values for Cr (21.0%), Ni (12.9%), and Pb (11.5%). Cr and Ni have similar spatial distribution characteristics (Figure 6). Areas with high values of these HMs were situated in the mid-lower reaches of rivers, and there were more stainless steel processing plants in the middle rivers; stainless steel is rich in Cr and Ni [67]. Wastewater from industrial production is transmitted through rivers, resulting in the HM pollution of farmland irrigation. Therefore, S6 was interpreted as sewage irrigation.

## 4. Conclusions

In this study, a combination of kriging interpolation, and PMF modeling was used for source apportionment of HMs in topsoils from the Wuqing District. The geoaccumulation index and the pollution load index suggested that Cd, As, Pb, and Cu were the primary poisonous elements in soil, causing potential risks in a local environment, and Cd was the main factor causing ecological risks. By comparing the relationship between total and available HMs in soil, it was found that the proportion of DTPA Cd, As, and Cu was higher. Therefore, in order to study whether the accumulation of HMs in vegetables was related to higher available HMs, analysis of HM accumulation in vegetables by bioconcentration factor proved high transportation of Cd from soil to vegetables with the average content in vegetables slightly higher above the allowable threshold (0.02 mg·kg^−1^). Furthermore, the spatial distribution of HMs in soil shows that the pollution was non-homogeneous; high values of the metal elements were observed in the southwest of the study area where there were many metal manufacturing industries, indicating that human activities negatively affect the farmland soil environment.

Using correlation analysis and kriging interpolation, the spatial distribution and accumulation of HMs shows that Cd has a relatively concentrated spatial distribution in the area identifying its point source pollution characteristics; Zn and Pb have similar spatial differentiation characteristics with higher values concentrated near traffic-intensive roads and rivers. The distribution of Cr, Ni, and As were highly consistent with soil types. The spatial distribution of Cu pollution was more concentrated than other HMs, with a higher value of Cu concentrated in the northeast of Wuqing district. Also, using PMF models that combined the spatial distribution and accumulation analysis results based on the kriging interpolation, the source apportionment showed that Cd might be derived from industrial activity; As and Pb might be ascribed to agricultural practices; Ni, Cu, Cr, and As mainly originated from natural sources; Zn and Cu were from organic fertilizer; Pb and Cd mainly came from traffic discharge; and Cr, Ni, and Pb were attributed to sewage irrigation.

## Figures and Tables

**Table 1:** (a) *I_geo_* value and assessment standards; (b) Potential ecological risk index and assessment standards; (c) Ecological risk factor and assessment standards.

(a) *I_geo_*	Risk Grade	(b) *PLI*	Risk Grade	$(c)\;E_{r}^{i}$	Risk Grade
*I_geo_* < 1	Uncontaminated to Medium polluted	*PLI* ≤ 1	Low level of pollution	$E_{r}^{i}$ < 40	Low potential risk
1 < *I_geo_* ≤ 2	Medium polluted	1 < *PLI* ≤ 2	Moderate level of pollution	$40\;<\;E_{r}^{i}\leq \;$80	Moderate potential risk
2 < *I_geo_* ≤ 3	Medium to heavily polluted	2 < *PLI* ≤ 5	High level of pollution	$80<E_{r}^{i}\leq$ 160	Considerable potential risk
3 < *I_geo_* ≤ 4	Heavily polluted	*PLI* > 5	Extremely high level of pollution	$160<E_{r}^{i}\leq$ 320	High potential risk
4 < *I_geo_* ≤ 5	Heavily to extremely polluted			$320<E_{r}^{i}$	Serious
*I_geo_* > 5	Extremely polluted				

**Table 2:** Statistical results of soil properties and HMs concentrations (*n* = 92).

	Unit	Max	Min	Mean	Standard Deviation	Skewness	Kurtosis	CV (%)	Background Value ^a^	Risk Screening Values ^b^/Exceedance Rates (%)	
										6.5 < pH ≤ 7.5	pH > 7.5
pH		8.89	6.63	7.69	0.39	−0.04	0.46	5.01	/		
SOM	g·kg^−1^	42.48	7.17	19.30	6.27	0.85	1.98	32.48	/		
Cd	mg·kg^−1^	2.83	0.06	0.23	0.31	7.03	56.15	136.15	0.09	0.3 (6.25%)	0.6 (3.16%)
Pb	mg·kg^−1^	191.87	17.91	46.28	29.27	3.05	11.05	63.25	20.6	120 (0.0%)	170 (3.16%)
As	mg·kg^−1^	25.73	1.08	13.36	3.26	0.79	3.93	24.39	8.39	30 (0.0%)	25 (1.59%)
Cr	mg·kg^−1^	113.30	48.21	69.33	12.82	0.89	0.50	18.50	63.69	200 (0.0%)	250 (0.0%)
Cu	mg·kg^−1^	233.30	17.26	35.76	23.80	6.44	51.45	66.55	19.88	100 (6.25%)	100 (0.0%)
Ni	mg·kg^−1^	47.19	19.89	29.49	6.82	1.10	0.33	23.14	26.69	100 (0.0%)	190 (0.0%)
Zn	mg·kg^−1^	801.93	61.03	113.64	78.81	7.43	63.49	69.35	66.87	250 (6.25%)	300 (0.0%)

^a^ Soils background values in Tianjin City (GB15618-2018). ^b^ Risk control standard for soil contamination for agricultural soils in China (GB15618-2018).

**Table 3:** Content of HMs in organic fertilizer.

	Unit	Max	Min	Mean	SD	Variance	CV (%)	Standard
Cd	mg·kg^−1^	0.59	0.04	0.19	0.11	0.01	0.55	3
Pb	mg·kg^−1^	18.82	1.22	5.81	4.53	20.54	0.78	50
As	mg·kg^−1^	80.63	3.75	24.77	18.76	352.05	0.76	15
Cr	mg·kg^−1^	46.32	22.89	32.48	6.79	46.09	0.21	150
Cu	mg·kg^−1^	616.02	9.63	132.69	167.70	28124.15	1.26	/
Ni	mg·kg^−1^	16.60	2.88	10.58	4.32	18.68	0.41	/
Zn	mg·kg^−1^	1220.61	117.25	425.84	320.11	102467.52	0.75	/

Note: SD: standard deviation; CV: coefficient of variation. Standard values for different elements in irrigation water were obtained from NY525-2012.

**Table 4:** HM content in irrigation water.

	Unit	Max	Min	Mean	SD	Variance	CV (%)	Standard
Cd	μg·L^−1^	0.33	0.26	0.30	0.01	0.00	0.05	10.00
Pb	μg·L^−1^	0.45	0.21	0.27	0.06	0.00	0.23	200.00
As	μg·L^−1^	28.41	0.12	9.57	7.74	59.87	0.81	50.00
Cr	μg·L^−1^	4.00	0.24	1.03	0.71	0.50	0.69	100.00
Cu	μg·L^−1^	35.44	1.52	4.83	6.25	39.07	1.30	1000.00
Ni	μg·L^−1^	6.30	1.71	3.17	1.11	1.23	0.35	/
Zn	μg·L^−1^	16.77	3.32	6.46	2.66	7.09	0.41	2000.00

Note: SD: standard deviation; CV: coefficient of variation. Standard values for different elements in irrigation water were obtained from GB5084-2005 (MEPRC, 2004).

## Data Availability

The study did not report any data.
